# Pubovesical Complex-Sparing Under Hypothermia During Robotic-Assisted Laparoscopic Radical Prostatectomy: A Single-Institution Case Series

**DOI:** 10.3390/jcm14248759

**Published:** 2025-12-10

**Authors:** Chin-Heng Lu, Min-Che Tung, Chu-Shan Yuan, Yi-Sheng Lin, Li-Hua Huang, Wei-Chun Weng, Chao-Yu Hsu, Tang-Yi Tsao, Yen-Chuan Ou, Chia-Che Chang

**Affiliations:** 1 Division of Urology, Department of Surgery, Tungs’ Taichung Metroharbor Hospital, Taichung City 435403, Taiwan; chinhenglu@gmail.com (C.-H.L.); tungminche@gmail.com (M.-C.T.); tung12197@gmail.com (Y.-S.L.); hlh0215@gmail.com (L.-H.H.); wcweng27@gmail.com (W.-C.W.); jowyu@msn.com (C.-Y.H.); 2 Rong Hsing Translational Medicine Research Center, National Chung Hsing University, Taichung City 402204, Taiwan; 3 Doctoral Program in Translational Medicine, National Chung Hsing University, Taichung City 402204, Taiwan; 4 Department of Nursing, Jen-Teh Junior College of Medicine, Nursing and Management, Miaoli 356006, Taiwan; 5 Division of Urology, Department of Surgery, Li’s Hospital Daja, Taichung City 437704, Taiwan; yuanchushan66@hotmail.com.tw; 6 Departments of Pathology, Tungs’ Taichung Metro Harbor Hospital, Taichung City 435403, Taiwan; tsao0425@yahoo.com.tw; 7 Department of Life Sciences, National Chung Hsing University, Taichung City 402202, Taiwan; 8 Graduate Institute of Biomedical Sciences, Rong Hsing Translational Medicine Research Center, The iEGG and Animal Biotechnology Research Center, National Chung Hsing University, Taichung City 402202, Taiwan; 9 Department of Medical Laboratory Science and Biotechnology, Asia University, Taichung City 413305, Taiwan; 10 Department of Medical Research, China Medical University Hospital, Taichung City 404327, Taiwan; 11 Traditional Herbal Medicine Research Center, Taipei Medical University Hospital, Taipei 110301, Taiwan

**Keywords:** robotic urological surgery, clinical outcomes, surgical techniques, patient-reported outcomes

## Abstract

**Background/Objectives:** Functional continence and potency outcomes are paramount for the pentafecta of robotic-assisted laparoscopic radical prostatectomy (RARP). We describe a modified approach of the pubovesical complex (PVC)-sparing technique under hypothermia for better continence and potency preservation. **Methods:** This is a retrospective single-institution case series. Thirty-three PVC-sparing RARP procedures under hypothermia were performed in patients with clinically localized prostate cancer by the same experienced surgeon. The method includes four principles: (1) modified PVC-sparing technique, according to Richard Gaston et al., (2) the use of near-infrared fluorescence technology and indocyanine green to identify the benchmark artery of the neurovascular bundle and blood supply for the PVC, (3) accessory pudendal artery preservation, and (4) hypothermia to reduce tissue edema. Functional outcomes, including continence, potency, and other surgical findings, are presented. This is a feasibility case series, not a comparative or hypothesis-testing study. **Results:** This study enrolled 33 cases from 15 April 2020 to 31 December 2022. Four patients had positive surgical margins. The urinary continence rate was 100% after Foley removal at a mean of 6.6 days. The potency rate was 74% (17/23) at 6 months and 91.3% (21/23) at 12 months. The inclusion of a small sample of patients from a single hospital and the selection of patient conditions were the study limitations. **Conclusions:** The modified approach we described is technically feasible, and it can expedite the restoration of urinary function and potency preservation. No severe complications occurred, and patients achieved good oncological outcomes.

## 1. Introduction

The prevalence of prostate cancer (PCa) is high in the USA and Europe [1]. In Asia, a persistent and rapidly increasing trend in the incidence and mortality rates of PCa has been observed since 1979 [2]. Radical prostatectomy (RP) is the standard of care for long-term treatment and control of PCa, and the number of RPs has increased [3].

As life expectancy increases, functional outcomes, especially urinary incontinence and erectile dysfunction, are important factors for treatment strategy selection. Patel et al. presented pentafecta for reporting RP outcomes, including freedom from perioperative complications, continence, potency, negative surgical margin, and freedom from biochemical recurrence [4]. With the neuroanatomical and physiological studies of continence and potency, precise identification and preservation of the neurovascular bundles (NVBs) and internal and external urinary sphincters were essential to preserve continence and potency after RARP. In practice, continence commonly returns in 1–6 months, and potency returns in 3–24 months [5].

Many surgical methods have been developed for RARP [6]. Dr. Richard Gaston reported his pubovesical complex-sparing RARP (PVC-RARP) approach in 2010 [7]. This method can well preserve the detrusor apron (DA), other PVC structures, and bladder neck; thus, the difficult posterior approach to Retzius-sparing RARP can be avoided.

Our team published the first series of 30 cases of RARP in Taiwan [8] and has accumulated over 2000 RARP cases [9]. This article reported a modified PVC-RALP, a revamped version of Dr. Richard Gaston’s original method [7], and a part of the procedure modified from our previous papers [10].

This technique involves (1) complete periprostatic anatomy preservation, (2) prostate or capsular artery preservation (using near-infrared fluorescence [NIRF] technology in conjunction with intravenous indocyanine green [ICG] to identify the “landmark artery” to preserve the main blood supply and the NVB), (3) the preservation of accessory pudendal arteries (APAs), and (4) localized hypothermia to reduce thermal injury-related tissue edema, suppress inflammation, and avoid peripheral nerve injury. Studies have proposed that local tissue cooling devices such as the endorectal cooling balloon (ECB) [11] can decrease thermal injury and secondary inflammation [11,12]. In our technique, we replaced the ECB with ice bags and an irrigation system to decrease the temperature of the surgical field.

This study aimed to describe the steps of the “modified PVC-sparing technique under hypothermia” and to report functional and oncologic outcomes.

## 2. Materials and Methods

### 2.1. Patient Selection and Eligibility Criteria

This study was designed as a single-institution retrospective case series aiming to evaluate the feasibility and outcomes of a modified pubovesical complex (PVC)-sparing technique under localized hypothermia during robotic-assisted laparoscopic radical prostatectomy (RARP).

The primary objective was to enhance functional recovery—specifically urinary continence and potency—while maintaining oncological safety. The rationale for combining PVC-sparing with hypothermia was based on prior evidence that periprostatic temperature reduction can attenuate tissue inflammation and nerve injury, thus accelerating postoperative recovery.

All operations were performed by the same surgeon following a standardized protocol to ensure reproducibility. Detailed records of intraoperative steps, temperature monitoring, and ICG usage were maintained.

To facilitate replication, we describe each surgical step with key landmarks and instrument settings: monopolar energy at 30 W, bipolar coagulation at 20 W, and continuous irrigation of cold saline (4 °C) throughout dissection. Ice packs were replaced every 15 min to maintain pelvic temperature between 24 and 26 °C, measured using a sterile digital probe.

Inclusion criteria: Clinically organ-confined disease (cT1–cT2) staged by CT and MRI scan, no lymph node or distal metastasis, normal preoperative continence.

Exclusion criteria: Neoadjuvant hormonal therapy, previous prostate, urethra, or bladder neck surgery, bilateral NVBs were not preserved, salvage RALP. Patients with anteriorly located tumors, as identified on preoperative MRI, were excluded.

Criteria for bilateral NVB preservation: Localized disease, the cancer was organ-confined, clinical stage T1 or T2. Preoperative MRI and CT imaging showed no evidence of capsular involvement or extraprostatic extension on either side of the prostate.

The study adhered to the PROCESS 2020 guideline for surgical case series reporting.

### 2.2. Data Collection

All patients were continent preoperatively. The preoperative erectile functions were evaluated and documented by the International Index of Erectile Function (IIEF)-5 score. Patients had to be completely continent (pad-free) and have an IIEF score ≥ 17 at baseline. Standard pertinent clinical data are shown in Table 1. Continence was defined as not using pads. Potency was defined as an IIEF score ≥ 17 with or without using phosphodiesterase 5 inhibitors (PDE5-Is). PSA or biochemical failure was defined as two serial serum PSA results > 0.2 ng/mL. All patients had a total follow-up duration of 2 years.

Functional outcomes, including continence and potency, were assessed prospectively by the surgeon, Dr. Ou.

### 2.3. Ethical Information

The study was performed in agreement with applicable laws and regulations, good clinical practices, and ethical principles, as described in the Declaration of Helsinki. This study was approved by the Institutional Review Board of Tungs’ Taichung Metroharbor Hospital (Approval no. 109007) (Approval Date: 15 April 2020) and ISRCTN (registration number ISRCTN10803536). The informed consent was waived by the Institutional Review Board of Tungs’ Taichung Metroharbor Hospital.

### 2.4. Localized Hypothermia

The ice pads used contained crushed ice placed in the digit parts of a surgical glove with a temperature of about 2–4 °C. The temperature around the prostate was about 35 °C without ice pads usage and decreased to 24–26 °C after ice pads usage. During RALP with monopolar and bipolar electrical cautery, the temperature can be as high as 42 °C without ice pads usage and decreased to 32–33 °C after ice pads usage. Moreover, a cold saline solution (4 °C) was also used. The ice pads were put around the cul-de-sac and bilateral pelvic wall and changed every 15 min, maintaining the temperature at 24 °C.

Intracorporeal temperature was measured using a 9F esophageal temperature probe. The probe was grasped by the robotic instrument and positioned within the operative field to obtain the temperature readings.

### 2.5. NIRF Technology in Conjunction with ICG

Here, 2 mL of ICG dye was injected intravenously during RALP, by which bilateral NVBs and PVC appeared green.

### 2.6. Da Vinci^®^ Surgical System

We used the da Vinci^®^ Xi robot system (Intuitive Surgical, Sunnyvale, CA, USA) with four multi-joint robotic arms: one controlling a 3D binocular endoscope and the other three controlling arms articulated instruments. Moreover, 0° and 30° lenses were used.

### 2.7. Surgical Procedure

#### 2.7.1. Patient Preparation, Positioning, Trocar Design, and Placement

**Figure 1:**
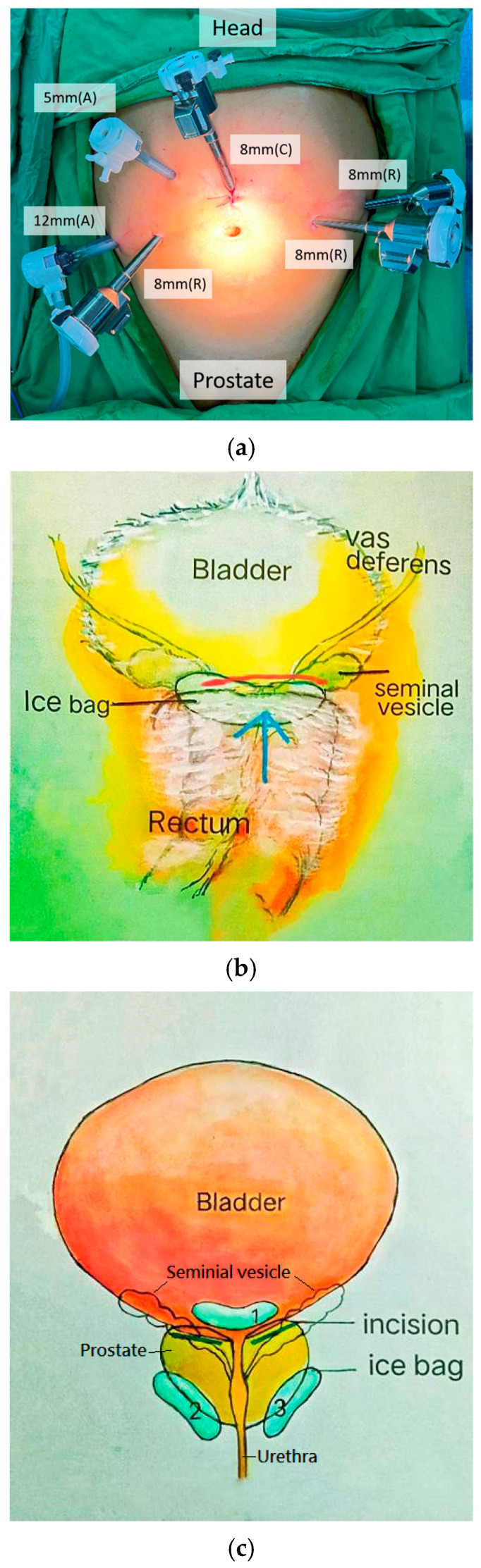
(**a**) Design and placement of the six laparoscopic trocars. A. assistant port; C. camera port; R. robotic port. (**b**) Incision of the posterior vesicorectal pouch and ice bag placement. (**c**) Ultradissection and ice bag locations. Ultradissection was started from the bilateral border of the dissected prostate edge and bladder neck to reach the seminal vesicles.

The patients were placed in the supine position, and the pressure points were padded. Shoulder pads and surgical tape were used to release pressure and secure the patient’s position. The steep Trendelenburg position of 20–30° was attempted before aseptic procedures. An 8 mm port was inserted above the umbilicus, and a pneumoperitoneum with CO_2_ gas insufflation up to 12–15 mmHg was created. The placement of the other five ports is shown in Figure 1a. The table was then maneuvered to a steep Trendelenburg position.

#### 2.7.2. Posterior Vesicorectal Pouch Approach for the Seminal Vesicle and Ice Pack Placement (Figure 1b)

The first step was the dissection of the seminal vesicle and vas deferens from the vesicorectal pouch (pouch of Douglas). A peritoneum incision was performed above the arcuate line of the peritoneal reflection at the level of the seminal vesicle. An ice bag was put into the vesicorectal pouch before the incision, and another ice bag was put into the space below the seminal vesicle after the incision. In low-risk PCa patients with a shallow risk of seminal vesicle involvement, the distal part of the seminal vesicles could be left in place. This maneuver reduces cavernous nerve injury and may improve continence and potency rates [7]. A cold knife dissection of the interfascial plane dissection was performed from the avascular plane between the prostatic fascia and Denonvilliers’ fascia and extended to the posterolateral aspect of the prostate [13].

#### 2.7.3. Dropping the Bladder and Creation of the Extraperitoneal Retzius Space

First, the urinary bladder was completely mobilized by an inverted U peritoneal incision above the level of the pubic symphysis. This incision extended to the level of the vas deferens on either side. The space anterior to the peritoneum and Retzius space was entered. The pre-prostatic fat was removed for lymph node dissection and better identification of the puboprostatic ligament and arcus tendineus.

#### 2.7.4. Lymph Node Dissection

After the risk evaluation of the patients, limited pelvic lymph node dissection was performed. The boundaries of the template are the obturator fossa, including the bladder at the medial aspect, the external iliac vein at the lateral aspect, the node of Cloquet at the inferior aspect, the bifurcation of the common iliac vessel at the superior aspect, and the obturator nerve at the posterior aspect. The APAs were identified and preserved.

#### 2.7.5. Lateral Approach: Bladder Neck Dissection from the Right Side

The lateral approach involved dissecting the edge inside the lateral aspect of the pubovesical ligaments and outside the junction of the detrusor of the bladder and prostate base. Ice bags were placed near the prostate border where the dissection began. We started the dissection on the right side, making a window from the right upper-lateral direction in the limit between the detrusor and prostate base, lateral to the pubovesical ligaments. Ultradissection was started from the bilateral border of the dissected prostate edge and bladder neck to reach the seminal vesicle. In some variations, no Denonvilliers’ fascia was present in the anterior of the seminal vesicles [14]. After dissection, a triangular space was created, which was formed by the prostate base, bladder neck, and right NVB and closed inferiorly by the seminal vesicle (Figure 1c). Then, the vas deferens and seminal vesicles were exposed and pulled out from the plane. The main arterial branches that supply the prostate base were ligated and clipped. The surgical field was ensured to begin the dissection of the NVB.

#### 2.7.6. Bladder Neck Dissection: Left Side

We created a window from the left upper-lateral aspect of the prostate base. The same procedure was performed for the right side and deep into the seminal vesicle and vas deferens. The vas deferens and seminal vesicles were pulled out from the plane.

#### 2.7.7. DA and PVC Preservation with ICG

This step focused on the PVC-sparing technique proposed by Gaston et al. in 2010 [7]. This technique maximizes the preservation of the PVC. The PVC included tissues at the ventral side of the prostate and connected to the urinary bladder and pubic symphysis, including the DA, pubovesical ligaments, dorsal vascular complex (DVC), Santorini’s plexus, and other tissues.

**Figure 2:**
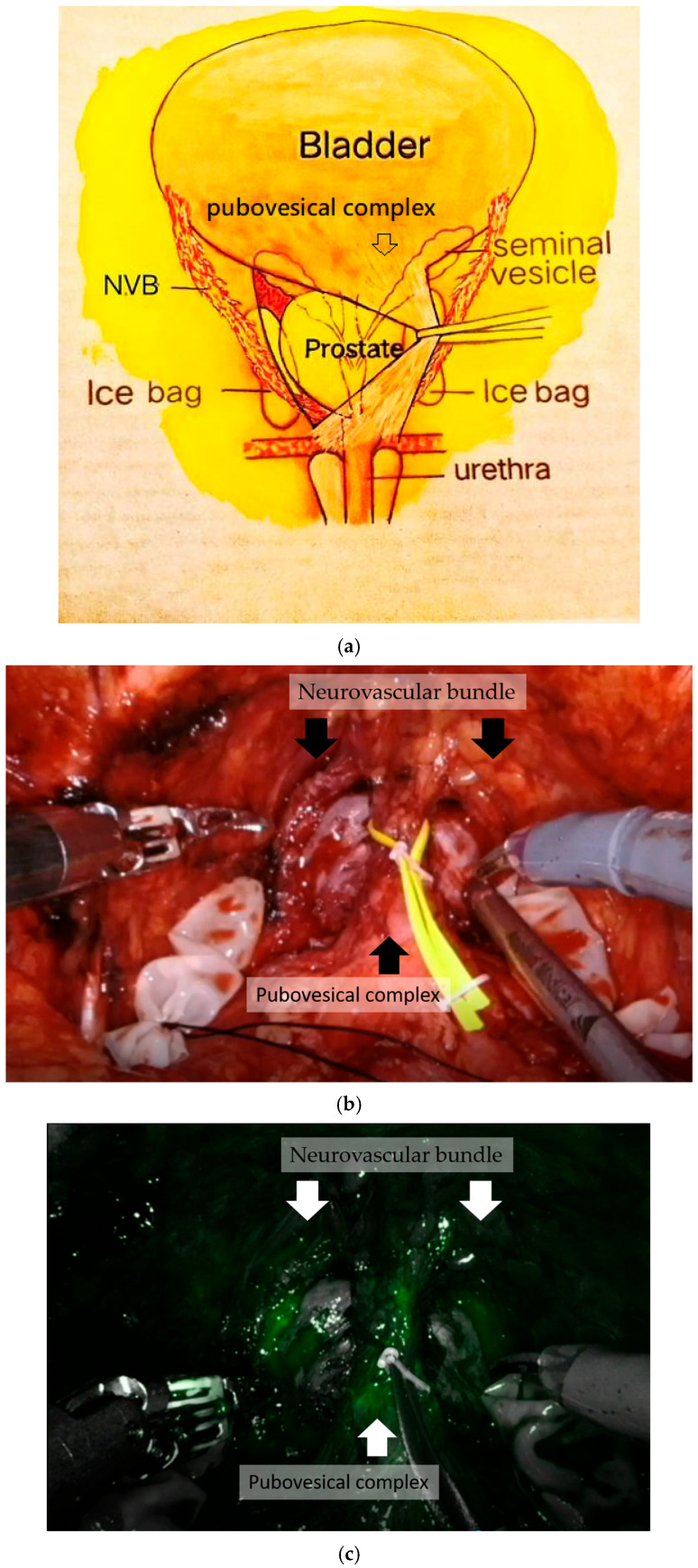

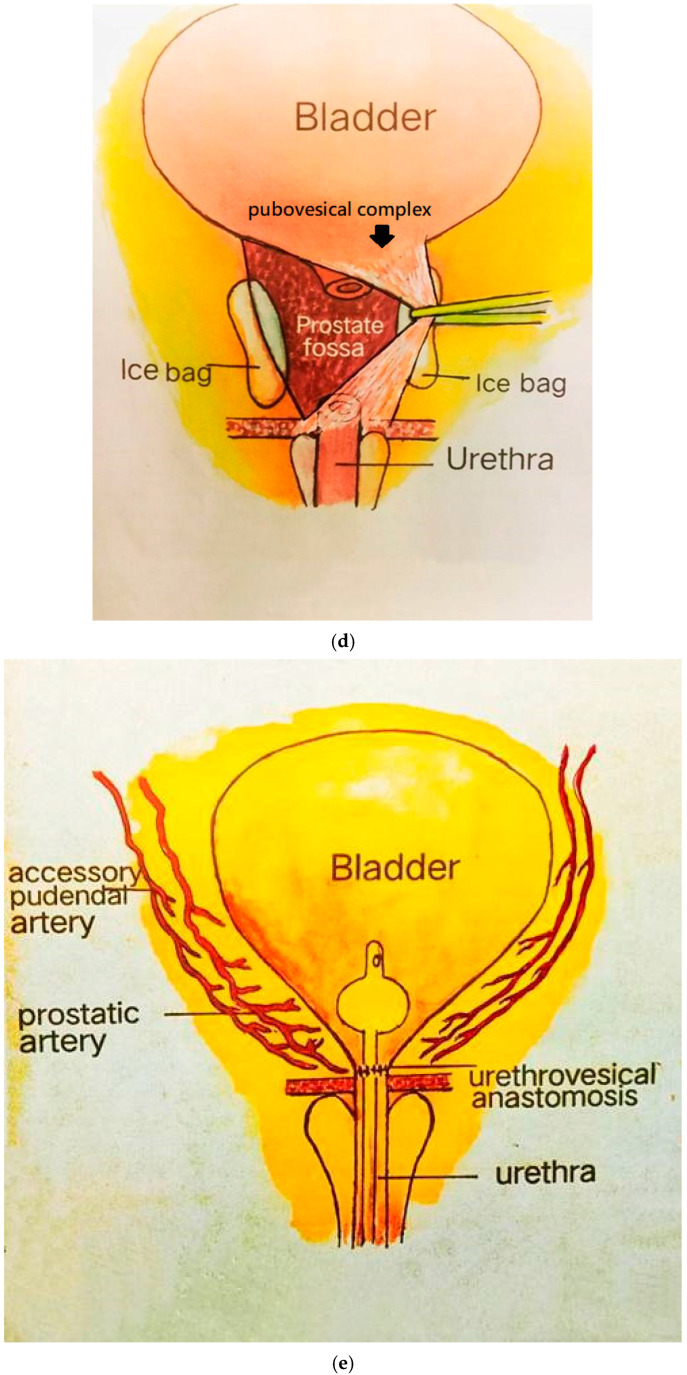
(**a**) The pubovesical complex was preserved and looped. (**b**) Neurovascular bundle and the pubovesical complex. (**c**) Neurovascular bundle and pubovesical complex identified by indocyanine green. (**d**) The urethra was divided, and the prostate and bilateral seminal vesicles were removed. (**e**) Urethrovesical anastomosis was performed by running method with two sets of 3-0 V-LOC™. Then, 200 mL of saline was injected to the Foley tube after anastomosis to confirm the absence of leakage.

We used ICG to identify the blood supply of the PVC. The fibromuscular tissue between the DVC and the prostate was dissected, and an avascular plane was found from the bladder neck to the anterior prostate–urethral junction. The separated PVC was looped and elevated (Figure 2a). The dissection procedure was performed under hypothermia to minimize neural and tissue damage (Figure 2b).

#### 2.7.8. Nerve-Sparing Technique: NVB Preservation with ICG

The NVBs are typically located at the bilateral rectolateral sides of the prostate (about 4/5 and 8/9 o’clock area). Recently, 1/5–1/4 nerve fibers were reported to distribute in the ventral prostatic capsule [15]. The endopelvic fascia adjacent to the prostatic fascia was incised at the position near 2 and 10 o’clock of the prostate. NIRF technology with ICG was performed to identify and determine the benchmark artery of the NVB and PVC (Figure 2c). All patients received bilateral full-sparing NVBs. When bilateral NVB was released, the posterior plane of the prostate was separated from Denonvilliers’ fascia. The bladder neck was preserved and divided carefully at the prostate base from the lateral side. At the end of the prostatectomy, the urethra was divided, and the prostate and bilateral seminal vesicles were removed (Figure 2d).

#### 2.7.9. Urethrovesical Anastomosis

Urethrovesical anastomosis was performed by the running method using two sets of 3-0 V-LOC™ (Figure 2e). Two sets of barded sutures were used beginning at the 5 o’clock site in clockwise and counter-clockwise directions continuously. To preserve the PVC, performing the anastomosis from the lateral view and passing the needle under the PVC is necessary. If the bladder neck is wide after prostate removal, bladder neck reconstruction from the 3 and 9 o’clock sites is performed before anastomosis.

After vesicourethral anastomosis, 200 mL of saline was injected into the Foley tube to confirm the absence of leakage. If extravasation was noted, the weak point was repaired immediately. A 15-F pelvic drain was placed via the left robotic port. The prostate and bilateral seminal vesicles were placed in an endobag and removed through the umbilical incision.

#### 2.7.10. Postoperative Care

The drainage tube was removed when the fluid volume was less than 100 mL/day or was stably decreasing without any complications. The Foley catheter was usually removed on day 7 before discharge. All the patients received PDE5-Is: initially, tadalafil 20 mg, one dose twice per week, followed by tadalafil 5 mg, one dose per day, 1 month after surgery.

#### 2.7.11. Histopathology

**Figure 3:**
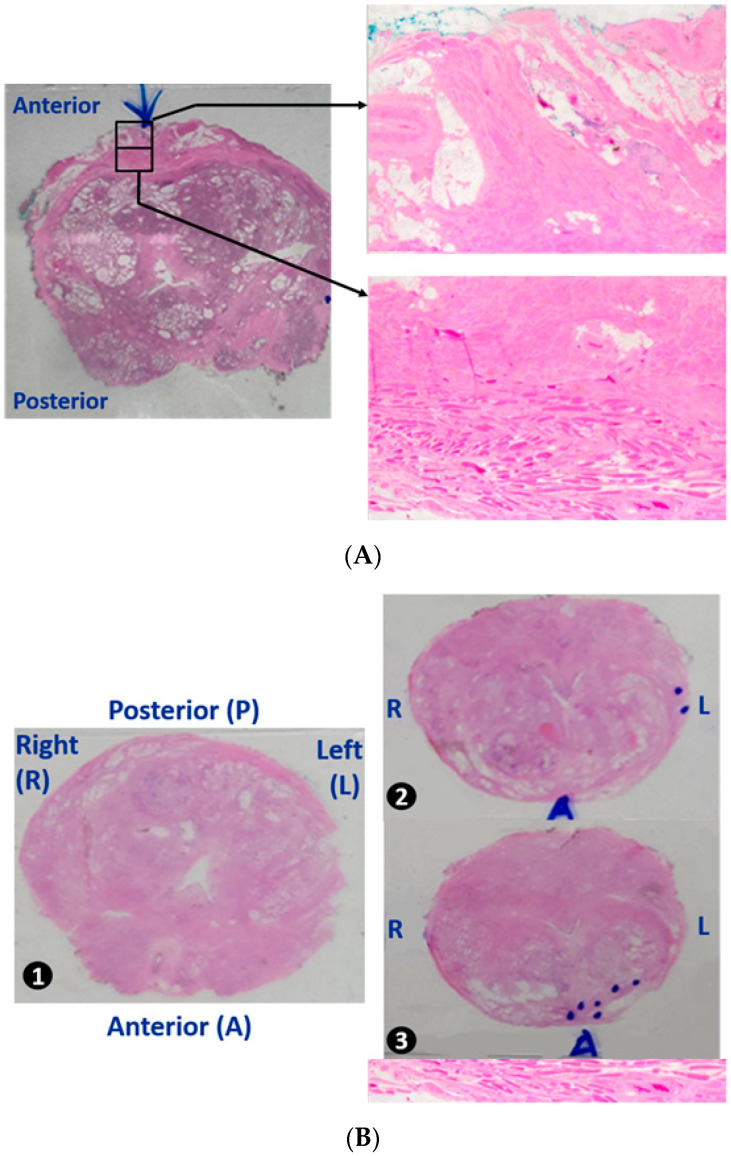
Final pathology: (**A**) standard dissection without PVC preservation and (**B**) modified PVC preservation. The lower part of the figure presents the microscopic view of the inserts. In (**A**), the arrow head is the PVC located at the anterior prostate, striated muscle (detrusor apron), fat tissue, small vessels, striated muscle fibers mingled with some smooth muscle fibers, and prostatic parenchyma with glands. (**B**) The PVC is preserved. Almost all striated muscular layers (DA) and the majority of fat tissues (represented by our marker during the anterior dissection) are not visible. PVC, pubovesical complex.

Figure 3A,B show the differences between the standard and PVC-sparing techniques in histologic specimens.

While the combination of PVC-sparing with localized hypothermia and ICG-guided dissection is intriguing, most components have been individually reported. The novelty lies in integration rather than discovery.

#### 2.7.12. Statistical Analysis

In this study, the Student *t*-test was used for continuous variables. All data were expressed as mean ± standard deviation. SPSS 20.0 for Windows (IBM, Chicago, IL, USA) was used for all the statistical analyses. A *p*-value of <0.05 was considered statistically significant.

This case series has been reported in line with the PROCESS Guideline.

## 3. Results

This study enrolled 33 cases from 15 April 2020 to 31 December 2022. The mean age was 64.61 ± 7.07 (mean ± standard deviation) (range, 50–80 years old). The mean BMI was 23.57 ± 2.82 (range, 17.7–29%). The mean console time was 128.76 ± 24.26 (range, 80–185) min. The mean blood loss was 94.85 ± 69.20 (range, 20–300). Complications occurred, including leg edema, skin allergy, and gouty arthritis in 2 out of 33 (6%) patients. The mean bladder catheterization time was 8 (range 7–9) days. The urinary continence rate was 27.3% (9/33) the day after Foley catheter removal, and 100% continence was achieved at a mean of 6.6 (range, 0–21) days after Foley catheter removal. To avoid overinterpretation, it is important to note that the reported “100% continence” rate is largely a consequence of the limited sample size and the selective nature of the included patient cohort. The potency rate was 74% at 6 months and 91.3% at 12 months. The mean tumor volume at pathology was 3.58 ± 4.37 (range, 0.13–19%) mL. The mean tumor percentage at pathology was 9.24 ± 12.3% (range, 0.2–61%). The mean specimen volume was 45.65 ± 23.52 (range, 21–119) mL. The PSM rate was 12% in 4 out of 33 patients. There were two cases of PSM < 1 mm and two cases of PSM > 1 mm. Potency assessment was performed in 23 of the 33 patients; the remaining 10 patients had preoperative IIEF-5 scores < 17 and were excluded from postoperative potency evaluation. We investigated potential predictors of postoperative continence and potency recovery. Among the examined variables, the only statistically significant factor was body mass index (BMI), with higher BMI being associated with a lower potency rate (Table 2 and Table 3). Table 4 further provides a comparative analysis between the present cohort and the series reported by Gaston et al., highlighting differences in patient selection and functional outcomes.

## 4. Discussion

Continence and potency are the most critical functional outcomes after RARP. In this study, we presented a modified pubovesical complex (PVC)-sparing technique performed under hypothermia to enhance both continence and potency preservation. The modification integrates four established surgical concepts: (1) PVC preservation, (2) near-infrared fluorescence (NIRF) technology with indocyanine green (ICG) for neurovascular bundle (NVB) identification, (3) accessory pudendal artery (APA) preservation, and (4) localized hypothermia to minimize tissue edema. These four principles have been previously described; however, our approach refines the hypothermic device setup and modifies several surgical steps to improve safety and reproducibility.

Over the past two decades, various surgical techniques and adjunctive devices have been developed to optimize outcomes in RARP [6]. The first RARP was reported by Binder and Kramer in 2001 [16] using an anterior approach. Subsequently, Bocciardi et al. introduced the posterior Retzius-sparing RARP (RS-RARP) in 2010 [17], performed through the Douglas space without bladder detachment. This approach minimized trauma to the anterior compartment—including the NVBs, Aphrodite’s veil, endopelvic fascia, Santorini’s plexus, pubourethral ligaments, and associated structures—and achieved a 96% continence rate within the first postoperative year [18]. In another RS-RARP study, continence recovery one week after catheter removal was 71%, compared with 48% for conventional RARP [19]. A meta-analysis by Checcucci et al. (2020) confirmed that RS-RARP provides significantly superior continence outcomes at 1, 3, and 12 months compared with the anterior approach [20]. Preservation of the PVC, which supports the external urethral sphincter and maintains the urethra in its natural pelvic position, likely facilitates earlier return of continence [7].

In 2007, Gaston et al. described a transperitoneal lateral approach to RARP [21]. This method began with the identification of the right prostate–vesicle angle, allowing dissection of the prostate base while preserving the dorsal vascular complex (DVC), detrusor apron (DA), and puboprostatic ligaments. The reported early continence rate was 80%, and the long-term complete continence rate reached 92.4%. Positive surgical margins (PSMs) occurred in 12.1% of pT2 and 29% of pT3 cases. However, the technique is technically demanding and not applicable to all patients.

In 2010, Gaston et al. introduced another PVC-sparing technique [7], combining DA and bladder neck preservation through a bilateral lateral approach. We compared the clinical characteristics and surgical outcomes of our series with those of Gaston et al., as summarized in Table 4. The results were comparable between the two techniques; our study achieved 100% continence at the time of Foley removal and 91.3% potency at 12 months postoperatively.

**Table 4:** Comparison between Ou’s series and Gaston’s series.

Series	Ou et al. N = 33	Gaston et al. N = 30
**Mean age yrs**	64.61 ± 7.07	52
**PSA ng/mL**	8.97 ± 4.90	7.15
**Clinical stage: ASAP/T1/T2**	2 (6%)/13 (39%)/18 (55%)	0/69.3%/27.9%
**Biopsy Gleason score ASAP/5/6/3 + 4/4 + 3/4 + 4**	2/0/8/17/3/13	0/4/26/0/0/0
**Pathology Gleason score** * ASAP/6/3 + 4/4 + 3/8/9	2/2/14/11/2/2	0/16/14/0/0/0
**Pathologic stage** * ASAP/T2a/2b/2c/3a	2 (6%)/10 (30%)/3 (9%)/15 (46%)/3 (9%)	4 (13.3%)/5 (16.6%)/18 (60%)/3 (10%)
**Positive surgical margin**	12% (4/33)	10% (3/30)
Continence **	9/33 = 27.3% (day 0), no incontinence (mean: day 6.6, 0–21)	80% (day 0), one pad 20% (day 0)
Potent ***	74% (17/23) in 6 months 91.3% (21/23) in 12 months (IIEF > 17)	73% (22/30) in 3 months (IIEF > 17)
**Erection Hardness Score (EHS)**	Score 3: 52.4% (11/21), Score 4: 47.6% (10/21)	Score 3: 59% (13/22), Score: 4: 41% (9/22)

* ASAP: atypical small acinar proliferation, ** Continence: day after removal Foley catheter, PSM: positive surgical margin, IIEF: International Index of Erectile Function. *** evaluate 23 patients, with phosphodiesterase type 5 inhibitors (13 patients), with vacuum constriction device (VCD) (two patients), with Nebido (one patient).

PSM remains an important consideration in evaluating surgical quality. A 2014 meta-analysis reported an average PSM rate of 15% across the RARP series [22]. Checcucci et al. (2020) observed a higher PSM rate in RS-RARP, particularly among patients with anteriorly located prostate cancer [20]. Lim et al. [23] and Eden et al. [24] reported PSM rates of 8% vs. 0% and 5% vs. 0% in RS-RARP compared with conventional RARP for anterior tumors in 2014 and 2018, respectively. Consequently, several investigators have cautioned against performing RS-RARP in cases of anteriorly located tumors [20]. Our technique also preserves the pre-prostatic components; therefore, patients with anteriorly located tumors were excluded from this series.

Most patients included in this study had low-risk prostate cancer, and their characteristics were comparable to those reported in the cohort described by Professor Ou [25]. In that study, the positive surgical margin (PSM) rate was 12.5%. All procedures in our study were performed by the same surgeon. Although certain refinements in surgical technique were implemented, the overall PSM rate did not increase.

There were four patients with positive surgical margins (PSMs). Their respective preoperative PSA levels, preoperative/postoperative Gleason scores, clinical/pathological stages, and tumor volume (mL)/prostate volume (mL)/tumor percentage (%) were as follows:

Case 1: 16.254 ng/mL, Gleason 8/7, T1c/T2b, 50 mL/15 mL/30%;
Case 2: 14.92 ng/mL, Gleason 7/7, T2b/T3a, 31 mL/19 mL/61%;
Case 3: 7.07 ng/mL, Gleason 7/7, T1c/T3a, 21 mL/0.525 mL/2.25%;
Case 4: 10.378 ng/mL, Gleason 7/7, T1c/T2c, 31 mL/6.096 mL/19.58%.

In our cohort, all PSM cases exhibited pathological upstaging compared with their clinical stage. However, none of the preoperative variables—including PSA, Gleason grade, tumor volume, or tumor percentage—reliably predicted the occurrence of PSM. Patients with anteriorly located prostate cancer had been excluded preoperatively. Moving forward, a higher intraprostatic tumor percentage and MRI findings suggestive of a higher T-stage should be carefully evaluated before considering the PVC-preservation approach during RARP.

The primary goal of our surgical modification was to minimize three types of tissue injury: (1) direct mechanical trauma during dissection or traction, (2) thermal injury resulting in neurapraxia, axonotmesis, or muscle degeneration, and (3) secondary inflammatory damage following mechanical or thermal stress [12]. Releasing both NVBs before applying traction to the prostate helps reduce mechanical injury. Localized hypothermia can mitigate thermal and inflammatory damage when used with cooling devices such as endorectal cooling balloons (ECBs) [11,12,26,27], thereby potentially accelerating the return of continence [28].

Factors influencing postoperative potency include neurogenic, arteriogenic, venogenic, and psychogenic mechanisms [29,30]. Local hypothermia and the intraoperative use of ICG for NVB and APA preservation can reduce neurogenic, arteriogenic, and venogenic injury, thereby improving functional recovery.

The potential benefits of intraoperative regional hypothermia during robot-assisted radical prostatectomy (RARP) may be explained through several complementary physiological mechanisms. First, local cooling may attenuate neuroinflammation by reducing pro-inflammatory cytokine activity and leukocyte recruitment around the neurovascular bundles, thereby limiting secondary neural injury [11,31]. Second, hypothermia is known to decrease oxidative stress by suppressing mitochondrial ROS production and stabilizing mitochondrial membranes, reducing oxidative-induced apoptosis after surgical manipulation [32,33,34]. Third, hypothermia may preserve microvascular perfusion by reducing endothelial permeability, preventing microvascular collapse, and improving capillary oxygenation in cooled pelvic tissues [5,35].

Fourth, by decreasing vascular leakage, regional hypothermia likely reduces tissue edema, thereby minimizing compression of the external urethral sphincter and cavernous nerves, which may facilitate earlier continence and potency recovery [5,11,35]. Fifth, the lowered temperature leads to metabolic suppression, decreasing ATP consumption, delaying ischemic injury, and reducing reperfusion-associated damage during traction and dissection of the neurovascular bundles [33,34]. Lastly, by reducing inflammatory, oxidative, and metabolic stresses simultaneously, hypothermia may create a microenvironment conducive to neuronal preservation and axonal regeneration, supporting faster recovery of functional pathways associated with continence and erectile function [5,11]. These integrated mechanisms align with clinical observations of improved functional outcomes in early hypothermia-assisted RARP cohorts, although randomized trials demonstrate variable efficacy, suggesting an interaction between cooling effectiveness and the magnitude of surgical trauma [36].

In our cohort, a higher BMI was associated with a lower rate of postoperative potency recovery. To contextualize this finding, we conducted a review of the relevant literature to evaluate whether similar associations have been reported in previous studies. Elevated BMI (overweight/obesity) is associated with technical difficulty (reduced workspace, more visceral/pelvic fat), longer operative times, and occasionally increased blood loss—factors that can compromise precision nerve sparing and therefore erectile outcomes; several cohorts identify BMI as a negative predictor of potency and a contributor to perioperative complexity that may delay continence recovery [37]. Meta-analytic evidence from 2020 to 2024 supports higher BMI as a negative predictor for both continence and potency after RARP [38].

This study is limited by its small sample size from a single institution, inclusion of only early-stage disease, and controlled prostate volume. Additionally, the learning curve for this method is longer than that for standard anterior RARP.

A potential limitation of this study is the possibility of selection bias, as the cohort primarily included patients with low-risk, small-volume tumors. This selective inclusion may have contributed to the favorable functional and oncologic outcomes observed, potentially overestimating the benefits of the procedure. Consequently, the generalizability of our findings to a broader patient population, including those with higher-risk or larger-volume tumors, may be limited. Furthermore, since all procedures were performed using a specific robotic surgical technique, caution should be exercised when extrapolating these results to other robotic approaches or to surgeons with differing levels of experience. Future studies with more diverse patient populations are warranted to validate the applicability of these outcomes across different risk groups and surgical techniques.

Nonetheless, our results demonstrate that the modified PVC-sparing technique under hypothermia is technically feasible and may accelerate postoperative functional restoration.

## 5. Conclusions

The modified pubovesical complex (PVC)-sparing robotic-assisted laparoscopic radical prostatectomy (RARP) performed under hypothermia is technically feasible and preserves continence and potency effectively (Supplementary Materials). Given the limited scale of this single-institution case series [39], larger multi-center and case–control studies are warranted to confirm these findings and evaluate long-term oncologic and functional outcomes.

Future work should include larger-scale validation and biomechanical modeling to optimize the degree and duration of cooling. Integration with robotic energy modulation and perfusion imaging may further refine this approach toward precision urologic surgery.

## Figures and Tables

**Table 1:** Retrospectively collected data.

**Preoperative data**
Age Body mass index (BMI) Preoperative IIEF-5 score, Erection Hardness Score, potency Preoperative PSA, Gleason score of the TRUS biopsy, clinical T-stage
**Intraoperative data**
Console time Intraoperative complications Bilateral nerve-sparing procedures
**Postoperative data**
Postoperative IIEF-5 score, EHS, potency (6, 12 months), postoperative continence days Postoperative PSA, prostate weight, Gleason scores, specimen volume, tumor volume, tumor percentage, PSM Catheterization time Complications
PSA = prostate-specific antigen; TRUS = transrectal ultrasounds; IIEF-6 = International Index of Erectile Function-6; PSM = positive surgical margins; EHS = Erection Hardness Score

**Table 2:** Risk factors analysis of continence at post-operation day 1.

	Day 1 Continence			Day 1 Incontinence			*p*
	N	Mean	SD	N	Mean	SD	
Age (years old)	9	63.78	6.83	24	64.92	7.27	0.827
BMI (%)	9	23.58	2.52	24	23.57	2.97	0.953
PSA	9	8.15	4.52	24	9.28	5.09	0.619
Specimen volume (mL)	9	41.44	22.86	24	47.23	24.05	0.207
Console time (min)	9	128.33	26.10	24	128.92	24.13	0.827
Blood loss (mL)	9	73.33	54.08	24	102.92	73.45	0.349

*p* for independent samples *t*-test. Body mass index (BMI), prostate-specific antigen (PSA).

**Table 3:** Risk factors analysis of potency post-operation.

	Potency			Impotence			*p*
	N	Mean	SD	N	Mean	SD	
Age (years old)	21	61.9	5.84	2	69.50	10.61	0.285
BMI (%)	21	24.45	2.15	2	20.33	0.70	0.016 *
PSA	21	8.93	5.80	2	10.25	0.51	0.332
Specimen volume (mL)	21	45.14	23.07	2	45.14	23.07	0.791
Console time (min)	21	132.38	21.25	2	115.00	7.07	0.285
Blood loss (mL)	21	108.10	75.14	2	50.00	0.00	0.332

*p* for independent samples *t*-test, * means *p* < 0.05. Body mass index (BMI), prostate-specific antigen (PSA).

## Data Availability

Data are unavailable due to privacy or ethical restrictions.

## Supplementary Materials

The following supporting information can be downloaded at https://www.mdpi.com/article/10.3390/jcm14248759/s1.
